# Correction for Bhavana et al., “A group B *Streptococcus* indexed transposon mutant library to accelerate genetic research on an important perinatal pathogen”

**DOI:** 10.1128/spectrum.03752-25

**Published:** 2026-02-26

**Authors:** Venkata H. Bhavana, Gideon H. Hillebrand, Kathyayini P. Gopalakrishna, Rebekah A. Rapp, Adam J. Ratner, Hervé Tettelin, Thomas A. Hooven

## AUTHOR CORRECTION

Volume 11, no. 6, e02046-23, 2023, https://doi.org/10.1128/spectrum.02046-23. During quality control screening, BEI Resources identified 23 wells in the original collection with suspected non-GBS contamination. New stocks of these samples were prepared from single GBS colonies (confirmed by ChromAgar and latex agglutination) and collected on a new partial 96-well plate #21. Plate #21 is publicly available with the rest of the library on the BEI Resources website, which also lists the original well positions of the contaminated stocks.

In the Supplemental Material, Suppl. Data 3, paragraph 4, lines 2–4: “Plus-strand...complement.” should read “R mapped insertions will have the transposon oriented in the 5′ to 3′ direction below, while F mapped insertions would demonstrate the reverse complement. The F- and R-mapping designation is relative to the chromosome, so it is independent of the orientation of the associated gene.”

## SUPPLEMENTAL MATERIAL

Suppl. Data 3 spectrum.03752-25-s0001.docx Recommendations for validation.

ASM does not own the copyrights to Supplemental Material that may be linked to, or accessed through, an article. The authors have granted ASM a non-exclusive, world-wide license to publish the Supplemental Material files. Please contact the corresponding author directly for reuse.

