# Supplementary material for: A group B Streptococcus indexed transposon mutant library to accelerate genetic research on an important perinatal pathogen
Source: Microbiol Spectr. 2023 Nov 7;11(6):e02046-23. doi: 10.1128/spectrum.02046-23 (PMC10714824; doi:10.1128/spectrum.02046-23)
Supplement: Suppl. Data 3 — Recommendations for validation. [file spectrum.02046-23-s0003.docx]

**Supplemental Data 3: Information for PCR verification of A909 mutant library strains**

Introduction

While transposon insertion sites in the A909 mutant library have been characterized by molecular biology and next-generation sequencing multiplex techniques described in the main article text, most strains have not been individually verified by PCR. We therefore recommend that researchers using the indexed library perform confirmatory PCR with primer pairs complementary to the transposon (forward primer) and to a nearby chromosomal sequence (reverse primer) with strains of interest.

Specific recommendations

Genomic DNA can be purified from GBS strain A909 using a variety of commercially available kits. For a small number of strains, we use the Qiagen DNEasy PowerSoil Pro Kit (cat. # 47014) on a spun pellet from a 5 mL overnight culture, extending the bead beating GBS lysis step to 25 minutes and otherwise following manufacturer instructions. For bulk extraction, we use a magnetic bead-based protocol such as the ThermoScientific MagMax Viral/Pathogen Ultra Nucleic Acid Purification kit (cat. # A42356), which can be performed manually or using automated liquid handling and/or multiplex magnetic purification devices.

Custom PCR primers can be designed using the full *Himar1* mini transposon sequence below in tandem with the GBS A909 genome sequence, which is available at: <https://www.ncbi.nlm.nih.gov/data-hub/genome/GCF_000012705.1/>

We use Tn Primer F1 (see below) as the transposon-complementary primer. Library users are advised to note the strand orientation of the transposon insertion. Plus-strand insertions will have the transposon oriented in the 5’ to 3’ direction below, while minus-strand insertions would demonstrate the reverse complement. We recommend using primers complementary to a distinct genomic site, well-separated from the transposon insertion, as positive controls to ensure that bacterial DNA extraction was successful and that PCR components are functional. Contamination controls with sterile water instead of genomic DNA template are recommended.

Colony PCR can be performed with strain A909, but is less reliable than PCR on purified genomic DNA.

*Himar1* mini-transposon sequence (1,435 bp)

(5’)TAACAGGTTGGATGATAAGTCCCCGGTCTAACAAAGAAAAACACATTTTTTTGTGAAAATTCGTTTTTATTATTCAACATAGTTCCCTTCAAGAGCGATACCCCTCGAATTGACGCGAATTCGCCCTTGATATCGAAGCAAACTTAAGAGTGTGTTGATAGTGCAGTATCTTAAAATTTTGTATAATAGGAATTGAAGTTAAATTAGATGCTAAAAATTTGTAATTAAGAAGGAGTGATTACATGAACAAAAATATAAAATATTCTCAAAACTTTTTAACGAGTGAAAAAGTACTCAACCAAATAATAAAACAATTGAATTTAAAAGAAACCGATACCGTTTACGAAATTGGAACAGGTAAAGGGCATTTAACGACGAAACTGGCTAAAATAAGTAAACAGGTAACGTCTATTGAATTAGACAGTCATCTATTCAACTTATCGTCAGAAAAATTAAAACTGAATACTCGTGTCACTTTAATTCACCAAGATATTCTACAGTTTCAATTCCCTAACAAACAGAGGTATAAAATTGTTGGGAGTATTCCTTACCATTTAAGCACACAAATTATTAAAAAAGTGGTTTTTGAAAGCCATGCGTCTGACATCTATCTGATTGTTGAAGAAGGATTCTACAAGCGTACCTTGGATATTCACCGAACACTAGGGTTGCTCTTGCACACTCAAGTCTCGATTCAGCAATTGCTTAAGCTGCCAGCGGAATGCTTTCATCCTAAACCAAAAGTAAACAGTGTCTTAATAAAACTTACCCGCCATACCACAGATGTTCCAGATAAATATTGGAAGCTATATACGTACTTTGTTTCAAAATGGGTCAATCGAGAATATCGTCAACTGTTTACTAAAAATCAGTTTCATCAAGCAATGAAACACGCCAAAGTAAACAATTTAAGTACCGTTACTTATGAGCAAGTATTGTCTATTTTTAATAGTTATCTATTATTTAACGGGAGGAAATAATTCTATGAGTCGCTTTTGTAGATATCAAGGGCGAATTCGCGTCCTCGGTACCGGGCCCCCCCTCGAGGTCGACATCCCTGGCTTGTTGTCCACAACCGTTAAACCTTAAAAGCTTTAAAAGCCTTATATATTCTTTTTTTTCTTATAAAACTTAAAACCTTAGAGGCTATTTAAGTTGCTGATTTATATTAATTTTATTGTTCAAACATGAGAGCTTAGTACGTGAAACATGAGAGCTTAGTACGTTAGCCATGAGAGCTTAGTA**CGTTAGCCATGAGGGTTTAGTTCG**TTAAACATGAGAGCTTAGTACGTTAAGCATGAGAGCTTAGTACGTGAAACATGAGAGCTTAGTACGTACTATCAACAGGTTGAACTGCTGATCTTCGGATCTACTGCATTTAATACTAGCGACGCCATCTATGTGTCAGACCGGGGACTTATCATCCAACCTGTT(3’)

Recommended transposon-binding PCR primer **Tn Primer F1** (bolded and underlined in full sequence above)

(5’) CGTTAGCCATGAGGGTTTAGTTCG (3’)
