# Supplementary figures and images for: A group B Streptococcus indexed transposon mutant library to accelerate genetic research on an important perinatal pathogen
Source: Microbiol Spectr. 2023 Nov 7;11(6):e02046-23. doi: 10.1128/spectrum.02046-23 (PMC10714824; doi:10.1128/spectrum.02046-23)

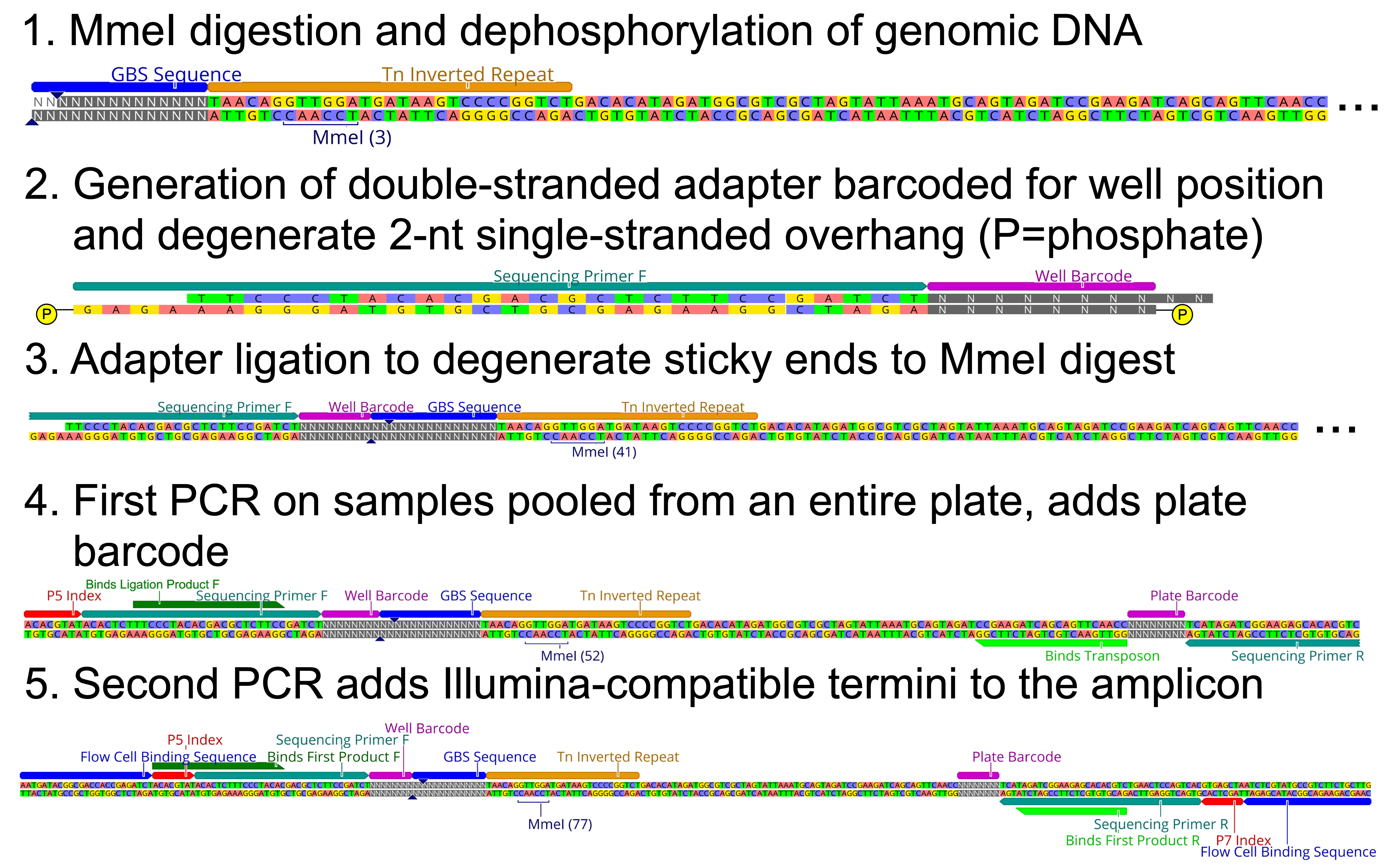

Supplement: Suppl. Figure 1 — Amplicon construction. [file spectrum.02046-23-s0004.tif]

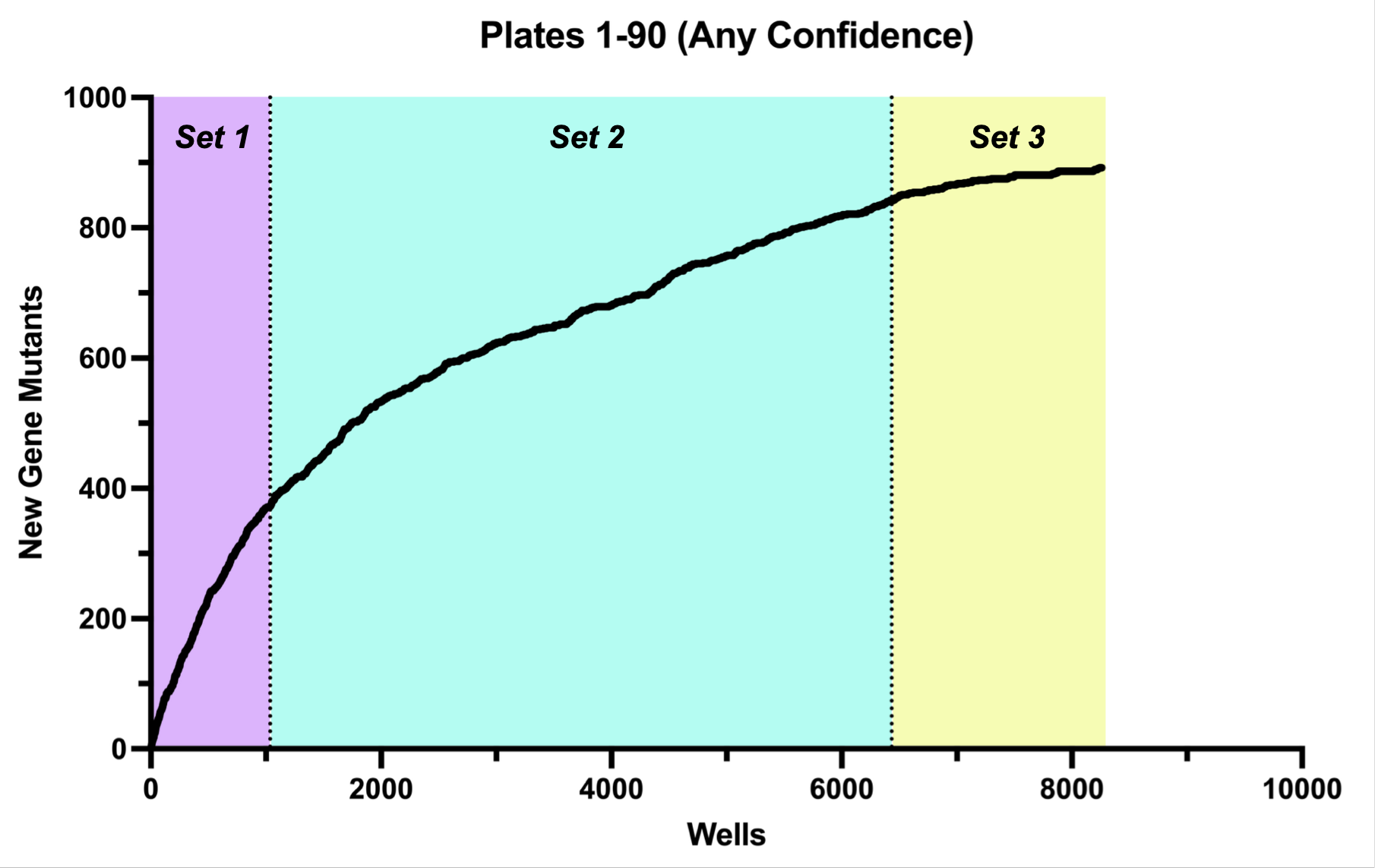

Supplement: Suppl. Figure 2 — New gene discovery rate. [file spectrum.02046-23-s0005.tif]

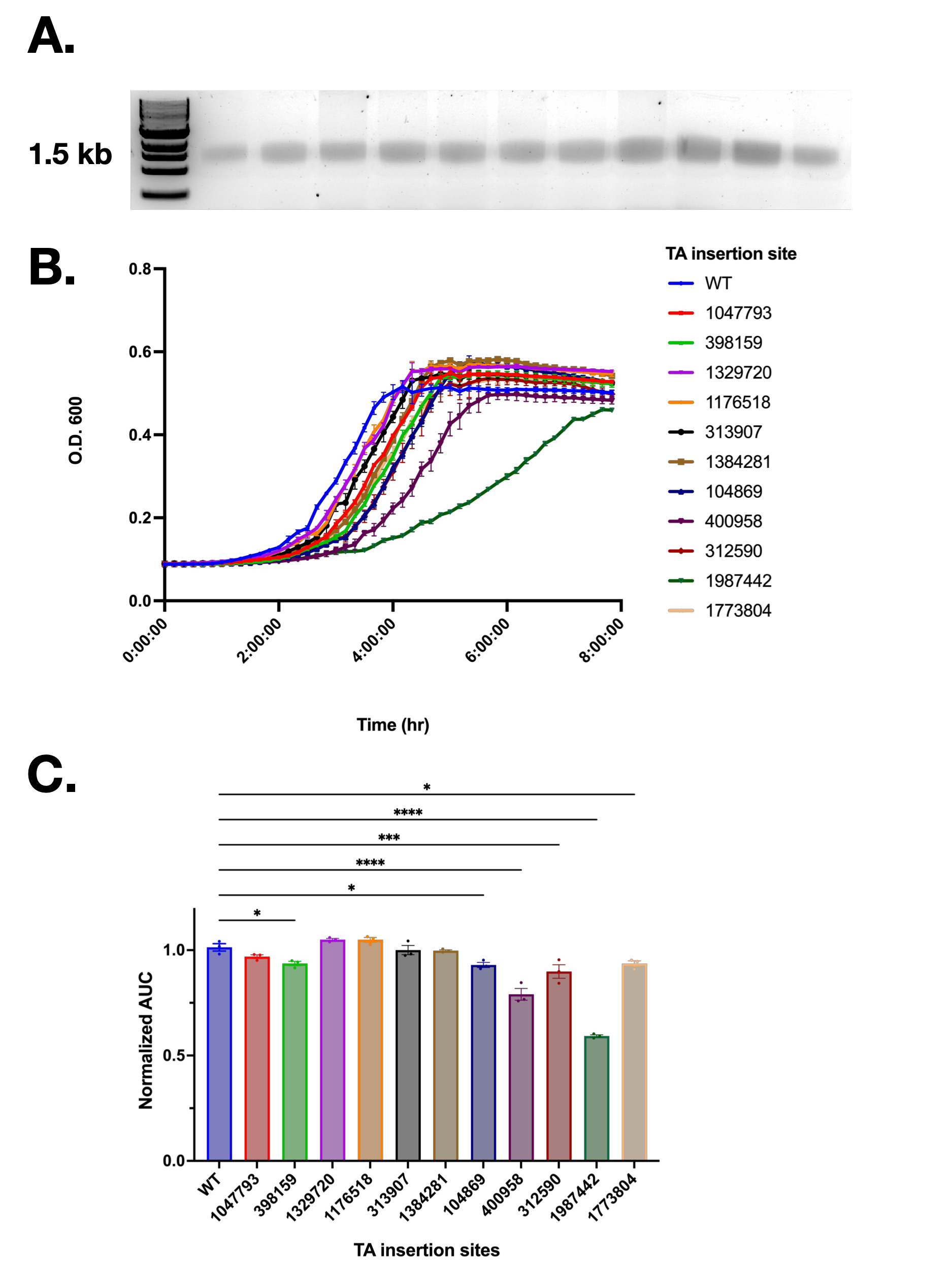

Supplement: Suppl. Figure 3 — Essential gene testing. [file spectrum.02046-23-s0006.tif]

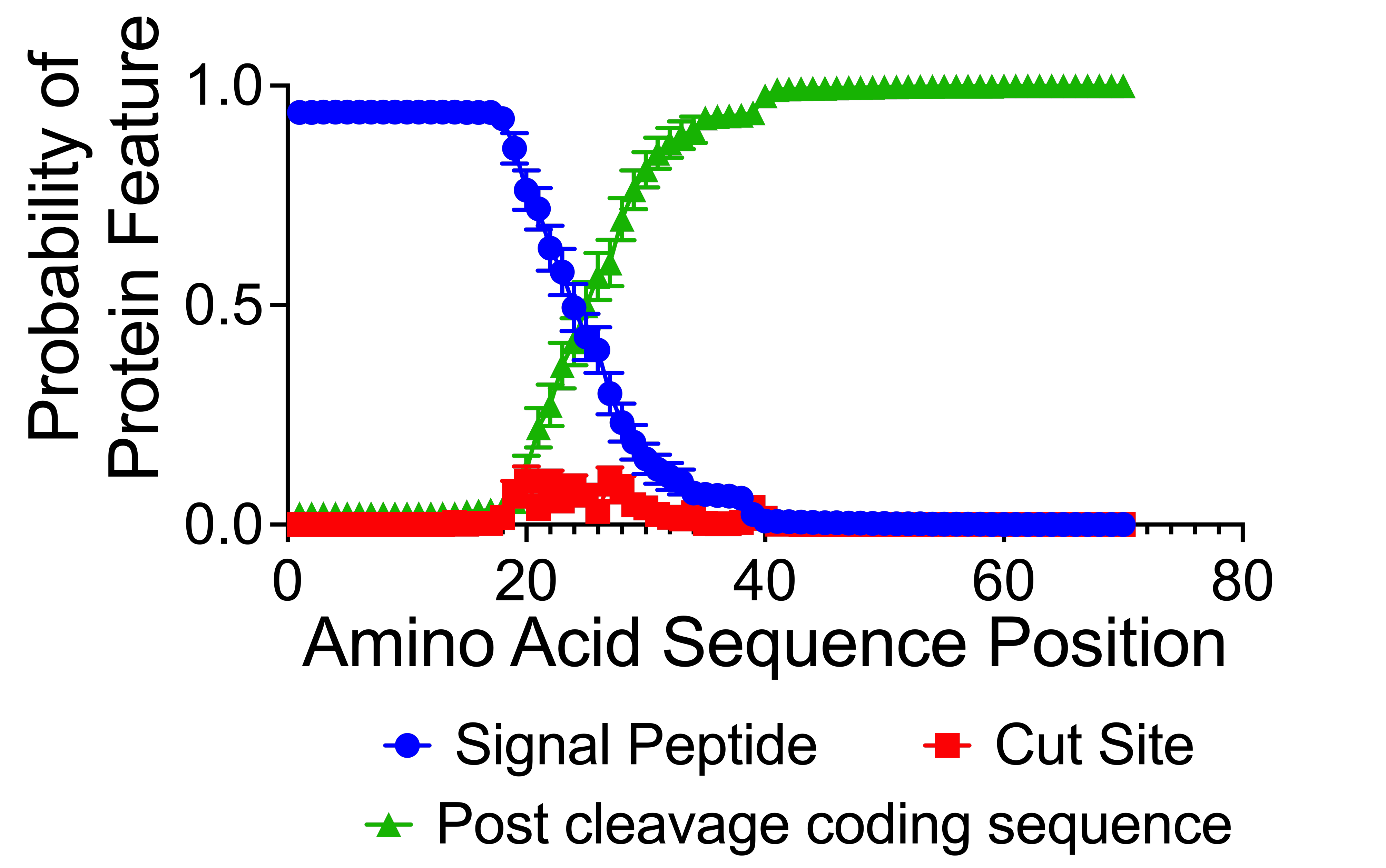

Supplement: Suppl. Figure 4 — Signal peptide analysis. [file spectrum.02046-23-s0007.tif]
