# Supplementary material for: A group B Streptococcus indexed transposon mutant library to accelerate genetic research on an important perinatal pathogen
Source: Microbiol Spectr. 2023 Nov 7;11(6):e02046-23. doi: 10.1128/spectrum.02046-23 (PMC10714824; doi:10.1128/spectrum.02046-23)
Supplement: Supplemental Data Captions — Aggregated captions for all supplemental figures and data. [file spectrum.02046-23-s0008.docx]

**Supplemental Data Information**

**Supplemental Figure 1: Steps used to generate dual-barcoded amplicons for sequence-based determination of transposon insertion sites.** The inverted repeat regions of the *Himar1* mini-transposon used for library construction bears a MmeI restriction site that results in cleavage 20-bp upstream, leaving a 16-bp region of adjacent GBS genomic DNA sequence (ending in a TA dinucleotide) with a 2-nt sticky end (**1**). 5’ and 3’ phosphorylated oligonucleotides barcoded for well position and with a partial Illumina sequencing primer binding site are annealed (**2**) and ligated to the MmeI digested transposon/GBS sequence terminus (**3**). A PCR reaction on pooled, well-barcoded DNA adds a plate identity barcode, an Illumina P5 index (for demultiplexing from a massively parallel sequencing run containing numerous libraries from different studies), and a partial reverse sequencing primer (**4**). A second PCR adds a P7 demultiplexing index, completes the reverse sequencing primer binding sequence, and adds flow cell binding sequences (**5**).

**Supplemental Figure 2: New gene transposon insertions as a function of well samples sequenced.** We performed three rounds of DNA sequencing (11 plates, 59 plates, 20 plates in sets 1, 2, and 3, respectively). The chart shows new insertions into distinct GBS genes as a function of each new well processed. The same gene identified multiple times—either as the same insertion site or as different insertions into the same gene—do not add to the tally in this figure.

**Supplemental Figure 3: Analysis of 11 essential gene mutant strains.** Genomic DNA from 11 essential gene transposon insertion mutant strains were screened by PCR to confirm expected insertions (**A**) using the same PCR strategy as in Fig. 4D**.** The 1.5 kb bands are in the same order as the strains are listed in panels B-C. Growth curves of triplicate replicates (**B**) show different growth profiles when compared to A909 WT strain when grown in TSB at 37°C for 8 hours. Area under the curves (**C**) for the growth curves in panel B (one-way ANOVA with Bonferroni correction for multiple comparisons; * p<0.05, *** p<0.005, **** p<0.001).

**Supplemental Figure 4: SignalP analysis of core GBS signal peptide-encoding genes.** SignalP was used to determine the probability of each amino acid (positions 1-70) being part of a signal peptide, cleavage site, or post-cleavage protein. Each point shows the mean probability for that amino acid position across the entire set of proteins, with error bars showing standard error of the mean.

**Supplemental Data 1: Oligo and PCR primer sequences**

**Supplemental Data 2: Complete library insertions and metrics.** TAcoordinate (column F) is the insertion site in the A909 genome while TAposition % (column M) shows how far into a gene coding sequence or intergenic region the TA position is located. WellMappedTotal (column O) indicates how many reads with the expected template and corresponding well and plate barcodes were demultiplexed. CoordinateTotal (column P) indicates how many reads mapped to the final assigned TA coordinate. SeqLength (column R) indicates the length of the decoded genomic DNA region. Fitness (column S) shows the log_2_ fold-change value for the gene based on original Tn-seq analysis of the A909 genome (40).

**Supplemental Data 3: Validation PCR instructions**
